# mTOR Inhibition Drives Mutation-Specific Remodeling of Lysosomal and Autophagic Pathways and GCase Activity in PBMC-Derived Macrophages from Patients with GBA1-Associated Parkinson’s Disease

**DOI:** 10.3390/cimb48050473

**Published:** 2026-05-01

**Authors:** Anastasia Bezrukova, Katerina Basharova, Anton Emelyanov, Anna Lavrinova, Anna Krapova, Ekaterina Galkina, Ekaterina Skudarnova, Galina Baydakova, Irina Miliukhina, Ekaterina Zakharova, Sofya Pchelina, Tatiana Usenko

**Affiliations:** 1 Petersburg Nuclear Physics Institute Named by B.P. Konstantinov of National Research Centre «Kurchatov Institute», 1, mkr. Orlova Roshcha, 188300 Gatchina, Russia; 2 Department of Molecular Genetic and Nanobiological Technologies, Pavlov First Saint-Petersburg State Medical University, L’va Tolstogo Str. 6-8, 197022 Saint Petersburg, Russia; 3 Research Center for Medical Genetics, Moskvorechie Str. 1, 140207 Moscow, Russia; 4 Institute of Human Brain RAS, Akademika Pavlova Street, 12A, 197376 Saint Petersburg, Russia

**Keywords:** Parkinson’s disease, *GBA1*, autophagy, GCase activity, mTOR, Torin 1, macrophages

## Abstract

To date, we and others have demonstrated that GBA1-associated Parkinson’s disease (GBA1-PD) exhibits hyperactivation of mTOR and impairment of mTOR-regulated autophagy. Our previous study showed that the degree of autophagy impairment depends on the type of *GBA1* mutation in peripheral blood mononuclear cell (PBMC)-derived macrophages. Moreover, the type of *GBA1* mutation (“mild”—e.g., p.N370S or “severe”—e.g., p.L444P) correlates with PD severity and may influence therapeutic response. Here, we investigated the dose-dependent effects of GCase inhibition by conduritol β-epoxide (CBE) in SH-SY5Y cells on mTOR signaling, as well as the effects of mTOR inhibition by Torin 1 on mTOR-dependent autophagy-related proteins, lysosomal morphology, and lysosomal hydrolase activities in PBMC-derived macrophages from PD patients carrying GBA1-L444P or GBA1-N370S mutations. CBE induced dose-dependent activation of mTOR signaling in SH-SY5Y, as evidenced by dose-dependent accumulation of p-RPS6 (Ser235/236). mTOR inhibition decreased Beclin-1 protein levels while increasing the LC3B-II/LC3B-I ratio, LC3B–lysosome colocalization, and lysosome number regardless of mutation type in PBMC-derived macrophages. However, Torin1 reduced p62 levels in GBA1-N370S-PD, whereas lysosomal size decreased in GBA1-L444P-PD. Interestingly, Torin 1 increased GCase activity in both patient groups. These findings suggest that mTOR inhibition restores GCase function and autophagy and may represent a potential therapeutic strategy for GBA1-PD.

## 1. Introduction

Parkinson’s disease (PD) is one of the most common multifactorial neurodegenerative disorders, characterized by the loss of dopaminergic neurons in the substantia nigra of the brain and by the accumulation and aggregation of α-synuclein, leading to the formation of Lewy bodies [1]. The most common genetically defined form of PD is associated with mutations in the *GBA1* gene (GBA1-PD). The *GBA1* gene encodes the lysosomal enzyme glucocerebrosidase (GCase). Biallelic mutations in *GBA1* lead to the rare autosomal recessive disorder Gaucher disease (GD), which is characterized by reduced GCase activity and accumulation of its substrates, glucosylceramide (GlcCer) and glucosylsphingosine (GlcSph). In turn, heterozygous or homozygous *GBA1* mutations represent a genetic risk factor for PD, increasing disease risk by 5–30-fold depending on the population [2,3,4,5]. The molecular mechanisms underlying GBA1-PD remain unclear. Previous studies, including our own, have demonstrated that mTOR signaling is disrupted in GBA1-PD [6,7,8,9,10,11,12]. However, GBA1-PD patients are genetically heterogeneous, carrying various types of *GBA1* mutations. Stratification *GBA1* mutations based on their phenotypic severity in GD classifies two groups: “severe” (most commonly p.L444P) and “mild” (most commonly p.N370S). Patients with GBA1-PD carrying the «severe» p.L444P/N mutation (GBA1-L444P-PD) present with earlier disease onset, more pronounced motor symptoms, and greater cognitive impairment compared with those carrying the «mild» p.N370S/N mutation (GBA1-N370S-PD) [5,13,14,15]. It can be plausible that the type of *GBA1* mutation influences the molecular mechanisms of PD, which are not yet fully elucidated. The mTOR kinase acts as a key negative regulator of autophagy, a cellular process responsible for the degradation of damaged organelles and misfolded proteins, including α-synuclein [16,17]. Consistent with this, our earlier work demonstrated that, while mTOR activation occurs in both GBA1-L444P-PD and GBA1-N370S-PD patients, the extent of autophagy disruption differs between the two genotypes in peripheral blood mononuclear cell (PBMC)-derived macrophages. Specifically, GBA1-N370S-PD shows potential autophagosome accumulation and impaired lysosomal degradation, whereas GBA1-L444P-PD exhibits enhanced autophagic flux, although lysosomal dysfunction is also present [12]. The mutation type–dependent effects of *GBA1* mutations on autophagy dysfunction have previously been demonstrated in induced pluripotent stem cell (iPSC)-derived dopaminergic (DA) neurons from GBA1-PD patients, as well as in neuroblastoma cell lines stably expressing *GBA1* mutations [18,19].

At present, inhibition of mTOR activity is regarded as a promising therapeutic strategy for the treatment of PD and GBA1-PD (NCT06612593, NCT04127578). Accordingly, we propose that therapeutic responses in GBA1-PD may be influenced by the type of *GBA1* mutation investigated in this study. Therefore, in the current study, we evaluated whether there is a relationship between the accumulation of GCase substrates resulting from dose-dependent reduction in its activity and the activation of mTOR signaling in the SH-SY5Y neuroblastoma cell line. Also, we assessed the impact of mTOR inhibition by the small molecule Torin 1 on autophagy, lysosome number and size, lysosomal hydrolase activity, and lysosphingolipid levels in PBMC-derived macrophages from GBA1-L444P-PD and GBA1-N370S-PD patients. Macrophages are widely used as a classical model to investigate the pathogenesis of GD and GBA1-related disorders due to their central role in lysosomal degradation and strong phagocytic activity [20,21].

## 2. Materials and Methods

### 2.1. Participants

A total of 13 patients with GBA1-PD and 17 neurologically healthy individuals were enrolled in the study. Of these, 8 patients with GBA1-PD were included in an investigation of the mTOR inhibitor Torin 1 in PBMC-derived macrophages. The demographic and clinical characteristics of the participants are summarized in Table 1. GBA1-PD patients were diagnosed at the Institute of the Human Brain RAS (Saint-Petersburg, Russia). All participants underwent a standard neurological examination, and the diagnosis of PD was based on previously published criteria [22]. Patients with GBA1-PD were identified through systematic screening of individuals with PD for the two major *GBA1* mutations, p.L444P and p.N370S, using allele-specific polymerase chain reaction (PCR) and PCR followed by restriction fragment length polymorphism analysis, as previously described [5,23]. *GBA1* mutations were classified as “severe” or “mild” according to the GD phenotype with which they are associated. The p.L444P mutation was considered a “severe” mutation, as it is typically linked to the neuropathic forms of GD (types II and III), whereas the p.N370S mutation was classified as a “mild” mutation, as it is commonly associated with the non-neuropathic form of GD (type I). Based on this classification, patients with GBA1-PD were stratified according to mutation severity for subsequent analyses. The control group consisted of individuals who were examined at the Consultative and Diagnostic Center of Pavlov First Saint Petersburg State Medical University (Saint Petersburg, Russia) and had no history of PD or other neurological disorders. Control participants were confirmed to be negative for the *GBA1* mutations p.L444P and p.N370S. All samples included in the study were randomly selected from the same geographic region. There were no statistically significant differences between the groups in terms of sex or age (*p* > 0.05). The study protocol was approved by the Ethics Committee of Pavlov First Saint Petersburg State Medical University (Saint Petersburg, Russia) (Approval No. 307, dated 20 October 2025).

### 2.2. PBMCs-Derived Macrophages

PBMCs were isolated from 24 mL of peripheral blood from each participant by density gradient centrifugation (Ficoll Solution, density 1.077; Biolot, Saint Petersburg, Russia) and differentiated into macrophages using macrophage colony-stimulating factor (M-CSF, 10 ng/mL; BioLegend, San Diego, CA, USA) in RPMI 1640 medium (Biolot, Russia) supplemented with 10% fetal bovine serum (FBS; Capricorn Scientific, Ebsdorfergrund, Germany) and 1% gentamicin (Biolot, Russia). Cells were incubated at 37 °C in a 5% CO_2_ atmosphere for 5 days, after which PBMCs-derived macrophages from GBA1-PD patients were treated with the mTOR inhibitor Torin 1 (Abcam, Cambridge, UK) (100 nM) for 24 h. Phenotypic maturation of PBMCs-derived macrophages was confirmed by light microscopy and flow cytometry using antibodies against CD14^+^ and CD68^+^ (eBioscience, San Diego, CA, USA) [21,24]. All PBMC-derived macrophages were grown in triplicate for each participant.

### 2.3. SH-SY5Y Neuroblastoma Cell Culture and Conduritol-B-Epoxide (CBE) Treatment

The neuroblastoma cell line SH-SY5Y was cultured in DMEM/F12/L-glutamine medium (PanEco, Moscow, Russia) supplemented with 10% FBS (Capricorn Scientific, Germany) and 1% gentamicin (Biolot, Russia) and incubated for 24 h at 37 °C in a 5% CO_2_ atmosphere. The culture medium was then replaced with DMEM/F12/L-glutamine (PanEco, Russia), supplemented with 3% FBS (Capricorn Scientific, Germany), 1% gentamicin (Biolot, Russia), and 10 μM retinoic acid (RA; Acros Organics, Geel, Belgium). Cells were cultured for an additional 72 h under the same conditions. After that, the medium was replaced with Neurobasal medium (PanEco, Russia) supplemented with L-glutamine and NeuroMax 50× supplement (PanEco, Russia). CBE (MedChemExpress, Monmouth Junction, NJ, USA) was added at concentrations of 25, 50, and 100 μM or cells were left untreated (control), and cultured for an additional 48 h. Each experiment was performed in three independent cell cultures.

### 2.4. Western Blot Analysis

PBMC-derived macrophages from each participant and SH-SY5Y neuroblastoma cell were lysed in RIPA buffer supplemented with protease and phosphatase inhibitors (Merk Millipore, Burlington, MA, USA). Total protein concentration was determined using the BCA Protein Quantitative Detection Kit (Servicebio, Wuhan, China). Equal amounts of protein were separated by SDS-PAGE (20% gel for LC3B and 12% gel for all other proteins), transferred onto polyvinylidene fluoride (PVDF) membranes (Bio-Rad, Hercules, CA, USA), and incubated overnight at 4 °C with primary antibodies: phospho-mTOR (Ser2448) (AF3308, Affinity Biosciences, Changzhou, China), phospho-RPS6 (Ser235/236) (AF3354, Affinity Biosciences, China), Beclin-1 (PAJ557Hu01, Cloud-Clone Corp, Wuhan, China), p62 (PAD198Hu01, Cloud-Clone Corp, China), LC3B (A19665, ABclonal, Wuhan, China), Cathepsin D (PAB280Hu01, Cloud-Clone Corp, China), and GCase (A19057, ABclonal, China) (all 1:1000). Membranes were then incubated for 1 h with the corresponding HRP-conjugated secondary antibody (FNSA-0004, FineTest, Wuhan, China, 1:5000), Protein signals were developed using the Clarity Western ECL Blotting Substrate (Bio-Rad, USA) and imaged with a ChemiDoc system using ImageLab software version 6.1 (Bio-Rad, USA). Protein levels were normalized to GAPDH (AC036, ABclonal, China; 1:15,000). Western blot data were analyzed using Fiji software (version 2.14.0/1.54f).

### 2.5. Autophagy and Lysosome Assessment by LysoTracker Immunofluorescence

PBMC-derived macrophages were incubated with LysoTracker Red DND-99 (50 nM, Thermo Scientific, Waltham, MA, USA) for 30 min, fixed with 4% paraformaldehyde (Sigma-Aldrich, St. Louis, MO, USA) for 30 min and washed with phosphate-buffered saline (Rosmedbio, Saint Petersburg, Russia) for 10 min. Cells were then blocked with 1% bovine serum albumin (Biolot, Russia) for 30 min and stained with primary anti-LC3B antibody (A19665, ABclonal, China; 1:500) for 60 min, followed by Alexa Fluor 488-conjugated secondary antibody (Jackson ImmunoResearch Laboratories, West Grove, PA, USA; 1:400) for 60 min. Imaging was performed using a Leica TCS-SP5 confocal microscope (Leica Microsystems GmbH, Wetzlar, Germany) and analyzed with Fiji software (version 2.14.0/1.54f). Quantification was performed separately for each sample, analyzing at least three fields of view with a minimum of 10 cells per field. Lysosome number and size were quantified as previously described [25], with particle size set to 0.2–5 µm^2^ and circularity set to 0.1–1.0. LC3 colocalization with LysoTracker Red DND-99 was assessed described in [26].

### 2.6. Enzyme Activities and Sphingolipid Concentrations

Dry blood spot (DBS) cards were prepared by pipetting 20 µL of PBMC-derived macrophages at a concentration of 2 × 10^6^ cells/mL onto each spot. The DBS cards were allowed to dry at room temperature in open air for 2 h and then stored at −20 °C until extraction. SH-SY5Y cells were lyophilized and then resuspended in Dulbecco’s phosphate-buffered saline. GCase activity and HexSph concentration were normalized to total protein concentration. The activities of GCase (EC 3.2.1.45; deficient in Gaucher disease), galactosylceramidase (GALC, EC 3.2.1.46; deficient in Krabbe disease), alpha-galactosidase A (GLA, EC 3.2.1.22; deficient in Fabry disease), and acid sphingomyelinase (ASMase, EC 3.1.4.12; deficient in Niemann-Pick disease types A and B), as well as their respective substrates HexSph—a mixture of glycosylsphingosine and galactosylsphingosine; lysosphingomyelin (LysoSM), and lysoglobotriaosylsphingosine (LysoGb3) were measured by liquid chromatography-tandem mass spectrometry, as described previously with modifications [21,27,28,29].

### 2.7. Statistical Analysis

Statistical analyses were performed using R software (version 4.3.2) with built-in and pre-installed packages. Data normality was assessed using the Shapiro–Wilk test. Pairwise comparisons between independent groups were conducted using the two-tailed Wilcoxon rank-sum test (Mann–Whitney U test), while comparisons between related groups were performed using the two-tailed Wilcoxon signed-rank test. Spearman’s rank correlation coefficient was used to assess associations between variables. A *p*-value < 0.05 was considered statistically significant. Clinical characteristics of the study participants are presented as the mean ± standard deviation, and experimental values are presented as median (minimum–maximum).

## 3. Results

The present study aimed to evaluate the dose-dependent effects of CBE on GCase activity, HexSph levels, and mTOR pathway activation in SH-SY5Y neuroblastoma cells. mTOR activation was assessed based on the protein levels of p-mTOR (Ser2448) and p-RPS6 (Ser235/236). Furthermore, the effects of inhibiting mTOR kinase activity with the selective small-molecule inhibitor Torin 1 were investigated focusing on autophagy, lysosome number and size, as well as on lysosomal hydrolase activities and lysosphingolipid levels in PBMC-derived macrophages from patients with GBA1-L444P-PD and GBA1-N370S-PD. Previous studies, including our own, suggest that the type of *GBA1* mutation may influence the phenotypic presentation of PD. In particular, patients carrying “severe” mutations tend to exhibit an earlier age at onset, an increased disease risk, and more pronounced motor and cognitive impairments compared with those harboring “mild” mutations [5,13,14,15]. These observations indicate a potential association between mutation type and therapeutic response in GBA1-PD.

### 3.1. Dose-Dependent Effects of CBE on GCase Activity, HexSph Level and mTOR Signaling

**Figure 1:**
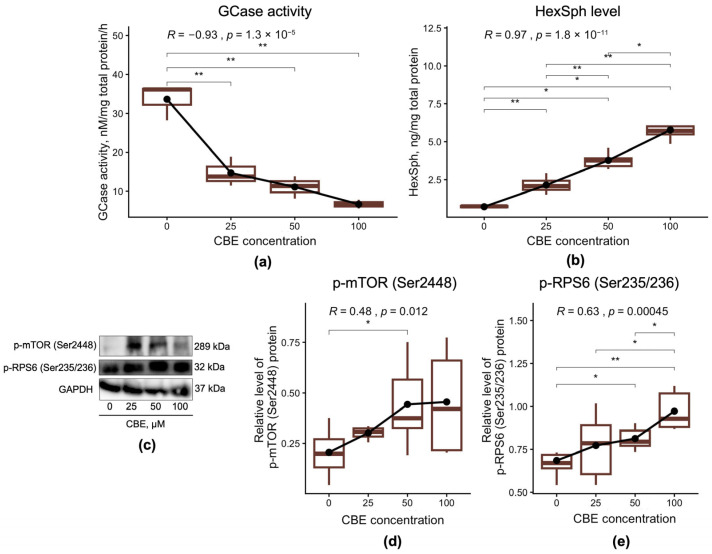
GCase activity, HexSph level and p-mTOR (Ser2448), p-RPS6 (Ser235/236) protein levels in SH-SY5Y cell line after dose-dependent CBE treatment: (**a**) GCase activity; (**b**) HexSph level; (**c**) Representative Western blot data; (**d**) p-mTOR (Ser2448); (**e**) p-RPS6 (Ser235/236). * *p* < 0.05; ** *p* < 0.01.

In the current study, we assessed the dose-dependent effects of CBE on GCase activity and HexSph levels in the SH-SY5Y neuroblastoma cell line (Figure 1). We also evaluated whether inhibition of GCase exerts a dose-dependent effect on mTOR signaling activation. The assessment was performed by measuring the protein levels of p-mTOR (Ser2448) and p-RPS6 (Ser235/236) in the SH-SY5Y cells (Figure 1c).

As expected, treatment of SH-SY5Y cells with CBE led to a dose-dependent decrease in GCase activity accompanied by a corresponding increase in HexSph levels across all tested concentrations (25, 50, and 100 μM) compared to untreated cells (GCase: *p* = 0.0062, 0.0041, and 0.0072, respectively; HexSph: *p* = 0.0095, 0.016, and 0.029, respectively) (Figure 1a,b). Interestingly, 100 μM CBE induced a greater accumulation of HexSph than 50 or 25 μM (*p* = 0.016, *p* = 0.0095, respectively), while 50 μM CBE also resulted in higher HexSph levels than 25 μM (*p* = 0.0043) (Figure 1b). At the same time, GCase activity negatively correlated with increasing doses of CBE (r = −0.93, *p* < 0.0001), whereas HexSph levels showed a positive correlation (r = 0.97, *p* < 0.0001) (Figure 1a,b).

In the context of altered GCase activity and HexSph levels, treatment with 50 µM CBE increased p-mTOR (Ser2448) levels (*p* = 0.023), while p-RPS6 (Ser235/236) levels increased in a dose-dependent manner at 50 and 100 µM (*p* = 0.022, *p* = 0.0022, respectively) compared to untreated neuroblastoma cells (Figure 1d,e). Moreover, 100 µM CBE induced higher p-RPS6 (Ser235/236) levels than 25 and 50 µM (*p* = 0.043, *p* = 0.035, respectively) (Figure 1e). A positive correlation was also observed between increasing CBE dose and the levels of p-mTOR (Ser2448) and p-RPS6 (Ser235/236) proteins (r = 0.48, *p* = 0.012, r = 0.63, *p* = 0.00045, respectively).

### 3.2. Effect of mTOR Inhibition by the Small Molecule Torin 1 on mTOR-Dependent Autophagy

**Figure 2:**
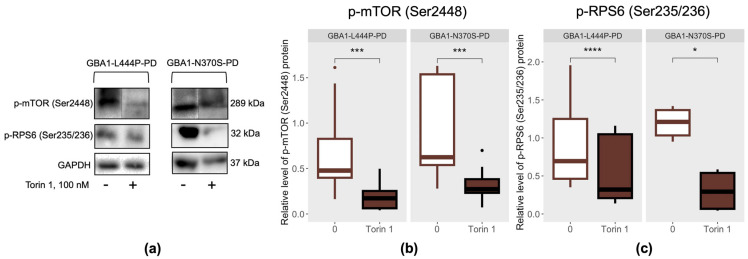
Efficacy of mTOR-signaling inhibition by the small molecule Torin 1 in PBMC-macrophages from GBA1-L444P-PD and GBA1-N370S-PD patients: (**a**) Representative Western blot data; (**b**) p-mTOR (Ser2448); (**c**) p-RPS6 (Ser235/236). * *p* < 0.05; *** *p* < 0.001; **** *p* < 0.0001.

The effectiveness of mTOR inhibition by the small molecule Torin 1 in PBMC-derived macrophages from GBA1-L444P-PD and GBA1-N370S-PD patients was evaluated by measuring phosphorylated mTOR (Ser2448, p-mTOR) and p-RPS6 (Ser235/236) levels (Figure 2a). Phosphorylation of mTOR at Ser2448 reflects its catalytic activity and activation of the mTOR complex, which regulates cell growth, metabolism, and protein synthesis. RPS6 is phosphorylated by S6K, a direct mTOR substrate, making p-RPS6 (Ser235/236) a downstream readout of pathway activity. Torin 1 treatment reduced in both p-mTOR and p-RPS6, confirming effective inhibition of the mTOR signaling cascade. Previously, we selected a Torin 1 concentration of 100 nM for assessment of its dose-dependent effects on mTOR signaling, autophagy, lysosomal hydrolase activity, and the levels of various forms of α-synuclein protein in PBMC-derived macrophages from healthy donors and SH-SY5Y cells [30].

In our previous study, patients with GBA1-L444P-PD and GBA1-N370S-PD were found to have increased levels of phosphorylated RPS6 (Ser235/236, p-RPS6) protein without changes in phosphorylated mTOR (Ser2448, p-mTOR) in PBMC-derived macrophages [12]. Here, Torin 1-mediated inhibition of mTOR activity reduced p-mTOR (Ser2448) and p-RPS6 (Ser235/236) levels in PBMC-derived macrophages from GBA1-L444P-PD and GBA1-N370S-PD patients (p-mTOR (Ser2448): *p* = 0.00043 and 0.00024, respectively; p-RPS6 (Ser235/236): *p* < 0.0001 and *p* = 0.031, respectively) compared with untreated cells (Figure 2b,c). These results indicate effective suppression of mTOR-signaling in patient-specific cells, independent of *GBA1* mutation type.

**Figure 3:**
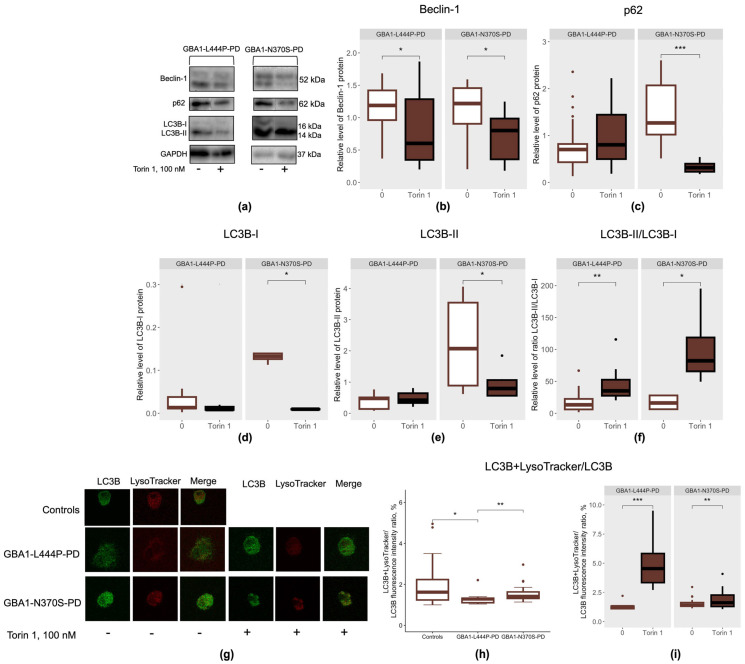
Autophagy in PBMC-derived macrophages from GBA1-L444P-PD and GBA1-N370S-PD patients after mTOR inhibition by Torin 1: (**a**) Representative Western blot data; (**b**) Beclin-1; (**c**) p62; (**d**) LC3B-I; (**e**) LC3B-II; (**f**) LC3B-II/LC3B-I; (**g**) Immunofluorescence data for LC3B (green) and lysosomes (LysoTracker) (red); (**h**) LC3B–lysosome (LysoTracker) colocalization; (**i**) LC3B–lysosome (LysoTracker) colocalization after mTOR inhibition. * *p* < 0.05; ** *p* < 0.01; *** *p* < 0.001.

The impact of mTOR inhibition by Torin 1 on autophagy was assessed by analyzing key autophagy-related proteins, including Beclin-1, involved in phagophore initiation and elongation; p62, which mediates the delivery of ubiquitinated cargo to autophagosomes; and LC3B, a central protein involved in autophagosome formation in PBMC-derived macrophages from GBA1-L444P-PD and GBA1-N370S-PD (Figure 3a). As LC3B exists in a cytosolic non-lipidated form (LC3B-I) and a lipidated autophagosome-associated form (LC3B-II), therefore, the LC3B-II/LC3B-I ratio is commonly used as an indicator of autophagic activity.

In our previous study, we demonstrated that patients with GBA1-L444P-PD exhibited increased Beclin-1 levels accompanied by reduced p62, without changes in LC3B-I, LC3B-II, or LC3B-II/LC3B-I ratios, whereas patients with GBA1-N370S-PD showed elevated Beclin-1, LC3B-I, and LC3B-II levels without changes in p62 or LC3B-II/LC3B-I ratios in PBMC-derived macrophages [12]. In the present study, we assessed LC3B colocalization with lysosomes using LysoTracker (Figure 3g) and found increased LC3B–lysosome colocalization in GBA1-L444P-PD patients compared to GBA1-N370S-PD patients and controls (*p* = 0.003, *p* = 0.014, respectively) (Figure 3h). The observed colocalization of LC3B with LysoTracker fluorescence may reflect autophagosome–lysosome fusion [31]. Taken together, these findings suggest that the type of *GBA1* mutation may contribute to differences in the degree of autophagy dysregulation observed in PBMC-derived macrophages from GBA1-PD patients.

Treatment with Torin 1 led to decreased Beclin-1 levels, an increased LC3B-II/LC3B-I ratio, and enhanced LC3B–lysosome colocalization (LysoTracker) in PBMC-derived macrophages from GBA1-PD patients, irrespective of mutation severity, compared to untreated cells (GBA1-L444P-PD: *p* = 0.015, 0.002, and 0.00024, respectively; GBA1-N370S-PD: *p* = 0.043, 0.031, and 0.005, respectively) (Figure 3b,f,i). Notably, only PBMC-derived macrophages from GBA1-N370S-PD patients demonstrated significant reductions in p62 levels upon Torin 1 treatment (*p* = 0.00049) compared to untreated cells (Figure 3c). These findings suggest that Torin 1 may effectively activate autophagic processes in PBMC-derived macrophages from GBA1-PD patients, with a more pronounced response observed in cells carrying the p.N370S mutation.

**Figure 4:**
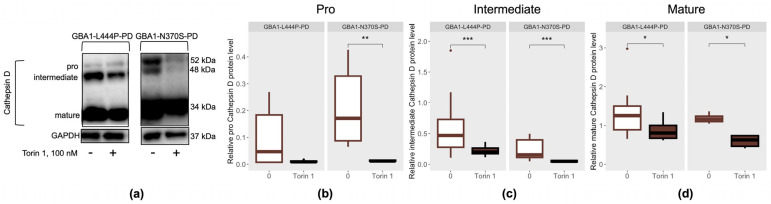
Cathepsin D in PBMC-derived macrophages from GBA1-L444P-PD and GBA1-N370S-PD patients after mTOR inhibition by Torin 1: (**a**) Representative Western blot data; (**b**) Pro form; (**c**) Intermediate form; (**d**) Mature form. * *p* < 0.05; ** *p* < 0.01; *** *p* < 0.001.

The effect of mTOR inhibition by Torin 1 on lysosomal proteolytic capacity was assessed by measuring the levels of the aspartic protease cathepsin D, which is involved in α-synuclein degradation [32] (Figure 4a). Cathepsin D exists in cells in three main forms. The pro-form is synthesized in the endoplasmic reticulum and matures in the Golgi apparatus; it is then processed into the enzymatically active intermediate form in endosomes, and is subsequently transported to lysosomes, where it acquires its mature form. In our previous study, we demonstrated that the intermediate form of Cathepsin D was elevated in patients with GBA1-L444P-PD, whereas patients with GBA1-N370S-PD exhibited increased levels of the pro form and decreased levels of the mature form in PBMC-derived macrophages [12].

In PBMC-derived macrophages from GBA1-PD patients, treatment with Torin 1 led to a decrease in the pro form of cathepsin D, specifically in cells carrying the p.N370S/N mutation (*p* = 0.0039), compared to untreated cells (Figure 4b). Additionally, PBMC-derived macrophages from GBA1-PD patients, regardless of mutation type, exhibited reduced levels of both intermediate and mature forms of cathepsin D in the presence of the Torin 1 inhibitor (GBA1-L444P-PD: *p* = 0.019, *p* = 0.00015, respectively; GBA1-N370S-PD: *p* = 0.031, *p* = 0.00098, respectively) compared to untreated cells (Figure 4c,d).

### 3.3. Effect of mTOR Inhibition by the Small Molecule Torin 1 on Lysosome Function

**Figure 5:**
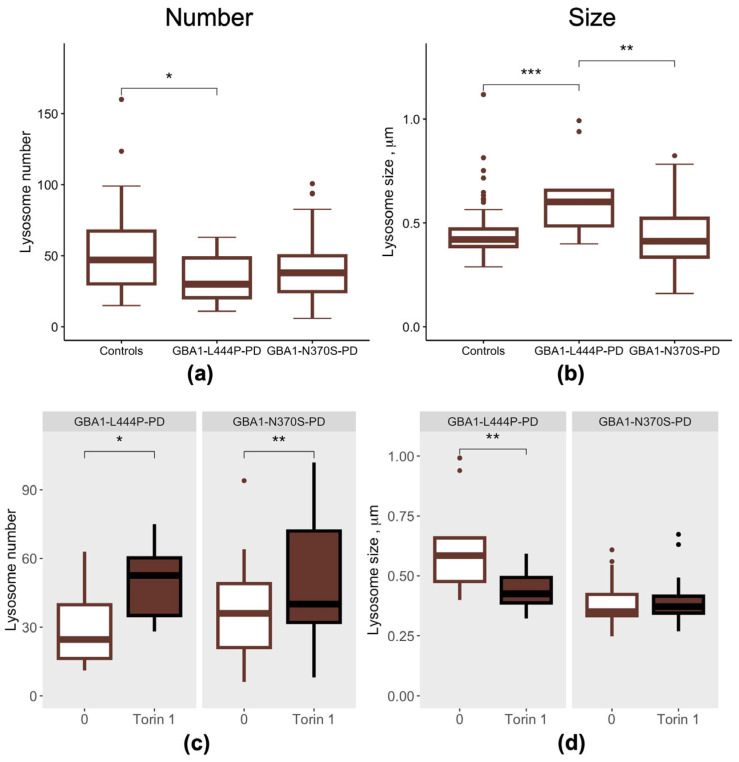
Lysosomes in PBMC-derived macrophages from GBA1-L444P-PD and GBA1-N370S-PD patients: (**a**) Lysosome number; (**b**) Lysosome size; (**c**) Lysosome number after mTOR inhibition by Torin 1; (**d**) Lysosome size after mTOR inhibition by Torin 1. * *p* < 0.05; ** *p* < 0.01; *** *p* < 0.001.

We next evaluated lysosome number and size in PBMC-derived macrophages from GBA1-PD patients according to mutation severity (Figure 5). GBA1-L444P-PD patients exhibited a reduced lysosome number compared to controls (*p* = 0.05) (Figure 5a), which was accompanied by an increase in lysosome size relative to both GBA1-N370S-PD patients and controls (*p* = 0.0022, *p* = 0.00081, respectively) (Figure 5b).

Following treatment of PBMC-derived macrophages with the mTOR inhibitor Torin 1, lysosome number increased in GBA1-PD patients regardless of *GBA1* mutation severity (GBA1-L444P-PD: *p* = 0.03; GBA1-N370S-PD: 0.0055, respectively) (Figure 5c), while lysosome size decreased in GBA1-L444P-PD cells compared with untreated cells (*p* = 0.0081) (Figure 5d).

**Figure 6:**
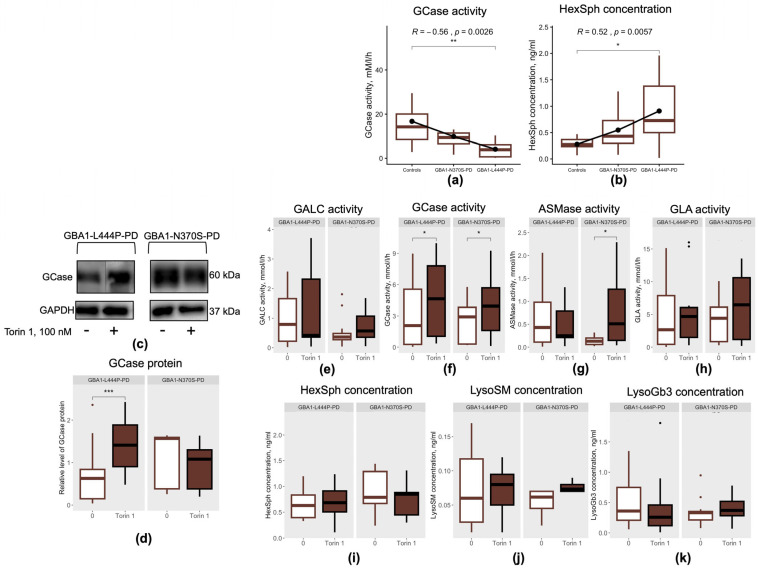
GCase protein level, lysosomal enzyme activities, sphingolipid concentrations in PBMC-derived macrophages from GBA1-L444P-PD and GBA1-N370S-PD patients: (**a**) GCase activity; (**b**) HexSph concentration; (**c**) Representative Western blot data; (**d**) GCase protein level; (**e**) GALC activity; (**f**) GCase activity; (**g**) ASMase activity; (**h**) GLA activity; (**i**) HexSph concentration; (**j**) LysoSM concentration; (**k**) LysoGb3 concentration. * *p* < 0.05; ** *p* < 0.01; *** *p* < 0.001.

Next, we assessed the effects of mTOR inhibition by Torin 1 on GCase protein levels (Figure 6c), GCase activity and its substrate lysosphingolipid HexSph (a mixture of GalSph and GlcSph, with GlcSph serving as the GCase substrate). In parallel, we evaluated the impact of Torin 1–mediated mTOR inhibition on the activity of other lysosomal enzymes involved in sphingolipid and ceramide metabolism, including GALC, ASMase and, GLA, as well as on their corresponding substrates HexSph (GalSph being the substrate for GALC), LysoSM and LysoGb3, which have also been implicated in the pathogenesis of PD [29,33,34,35,36]. In blood, we demonstrated that patients with GBA1-L444P-PD were characterized by reduced GCase activity and increased HexSph levels [37], whereas patients with GBA1-N370S-PD exhibited reduced GCase activity, elevated HexSph level together with increased activities of GLA, GALC and ASMase as well as elevated LysoGb3 levels [37,38]. In PBMC-derived macrophages, we also observed reduced in GCase activity (*p* = 0.0023) and increased in HexSph level (*p* = 0.023) in patients with GBA1-L444P-PD compared to controls (Figure 6a,b). The activities of other lysosomal hydrolases and the lysosphingolipids levels did not differ in GBA1-L444P-PD compared to controls (*p* > 0.05). At the same time, PBMC-derived macrophages from patients with GBA1-N370S-PD showed no alterations in the activities of lysosomal enzymes (GCase, GLA, ASMase, GALC) or in lysosphingolipid levels (HexSph, LysoGb3, LysoSM) compared to controls and GBA1-L444P-PD (*p* > 0.05). These results may suggest that PBMC-derived macrophages reveal functional defects only in p.L444P/N *GBA1* mutation, potentially due to their high lysosomal dependency, whereas whole blood analysis captures subtler biochemical alterations even in p.N370S/N mutations, likely reflecting cellular heterogeneity and systemic metabolic effects [39].

Interestingly, the type of *GBA1* mutations among PD patients was negatively correlated with GCase activity (r = −0.56, *p* = 0.0028) and positively correlated with HexSph levels (r = 0.52, *p* = 0.0057) in PBMC-derived macrophages (Figure 6a,b).

PBMC-derived macrophages from GBA1-L444P-PD patients cultured in the presence of the mTOR inhibitor Torin 1 exhibited increased GCase protein levels compared to cells cultured without the inhibitor (*p* = 0.00013) (Figure 6d). Torin 1 unexpectedly modulated GCase activity in patient-specific cells: mTOR kinase inhibition enhanced GCase activity in PBMC-derived macrophages from GBA1-PD patients regardless of the *GBA1* mutation type, compared to untreated cells (GBA1-L444P-PD: *p* = 0.023; GBA1-N370S-PD: *p* = 0.034, respectively) (Figure 6f). mTOR inhibition by Torin 1 also increased ASMase activity in PBMC-derived macrophages from GBA1-N370S-PD patients compared to untreated cells (*p* = 0.016) (Figure 6g). No significant changes were observed in GALC or GLA activities, or in the concentrations of the substrates HexSph, LysoSM, and LysoGb3 (*p* > 0.05) (Figure 6e,h–k).

## 4. Discussion

In this study, we evaluated the impact of dose-dependent pharmacological inhibition of GCase activity by CBE on GCase activity, HexSph concentration, and mTOR signaling in the SH-SY5Y neuroblastoma cell line. As well, we investigated the effects of mTOR inhibition by Torin 1 on key autophagy-related proteins, lysosome size and number, lysosomal enzyme activity, and lysosphingolipid concentrations in PBMC-derived macrophages from GBA1-PD patients stratified by mutation severity. We demonstrate that the degree of GCase activity reduction and HexSph accumulation in SH-SY5Y neuroblastoma cells correlates with mTOR signaling activation, as indicated by increased levels of its downstream target, p-RPS6 (Ser235/236). For the first time we show that mTOR inhibition differentially modulates autophagy and lysosome morphology according to *GBA1* mutation severity, while its capacity to enhance GCase activity is observed in all GBA1-PD patients regardless of mutation type.

mTOR kinase is a multifunctional protein that regulates various cellular processes, including growth, metabolism, and autophagy. In both in vitro and in vivo models of PD and GBA1-PD, omics-based and experimental studies have demonstrated that increased mTOR activity exacerbates α-synuclein accumulation and impairs autophagy [6,7,8,10,11,40,41,42].

We previously demonstrated that GBA1-PD is associated with hyperactivation of mTOR and impaired mTOR-regulated autophagy, with the degree of impairment depending on the type of *GBA1* mutation in PBMC-derived macrophages [12]. In GBA1-PD, we demonstrated that GCase activity and substrate accumulation do not differ between carriers of different mutation types [43], consistent with ours and other authors’ findings in blood [37,44,45]. Notably, an early Huh et al. showed that the type of *GBA1* mutation in PD was associated with GCase activity in a quantitative manner, with increasing severity of *GBA1* mutations linked to decreasing GCase activity in blood [46]. This finding was also confirmed in our study in PBMC-derived macrophages. In turn, several studies demonstrated that CBE-induced, dose-dependent reduction in GCase activity leads to a dose-dependent elevation of GlcCer in vitro and in vivo [47,48,49,50]. These findings were confirmed in our experiments using the SH-SY5Y neuroblastoma cell line, which showed a dose-dependent decrease in GCase activity and accumulation of HexSph (mix of GlcSph and GalSph) levels. A previous study by Srikanth et al. demonstrated that inhibition of GCase activity by CBE activates mTOR kinase, while different doses of GlcSph, a GCase substrate, induce a dose-dependent increase in mTOR activity in wild-type neurons [51]. Extending these findings, here we demonstrated that dose-dependent inhibition of GCase activity is associated with increased p-RPS6 (Ser235/236) levels in SH-SY5Y neuroblastoma cells, indicating activation of mTOR signaling. Thus, pharmacological inhibition of GCase, resulting in reduced enzymatic activity and substrate accumulation, activates mTOR signaling in SH-SY5Y cells, suggesting that targeting mTOR may represent a promising therapeutic strategy for GBA1-PD. This dose-dependent effect indicates that the severity of *GBA1* mutations—and the resulting extent of GCase deficiency and substrate accumulation—may influence therapeutic responses, particularly to mTOR inhibition in PBMC-derived macrophages from patients with GBA1-L444P-PD and GBA1-N370S-PD.

Currently, several classes of mTOR kinase inhibitors have been developed, targeting different components of the mTOR pathway. Among these, Torin 1 has demonstrated notable efficacy in iPSC-derived DA neurons from patients with GD and GBA1-PD, where it has been shown to reduce phosphorylated α-synuclein (Ser129) levels and restore autophagolysosomal function, highlighting its potential as a therapeutic approach for disorders characterized by GCase dysfunction [10,26]. However, such studies remain limited in number and do not account for the specific type of *GBA1* mutation. Here, we showed that mTOR inhibition by Torin 1 reduced p-mTOR (Ser2448) and p-RPS6 (Ser235/236) levels in PBMC-derived macrophages from GBA1-PD patients, irrespective of the *GBA1* mutation type, demonstrating effective suppression of the mTOR signaling.

Therapeutic strategies currently being developed for GBA1-PD primarily focus on enhancing GCase enzymatic activity, among which ambroxol is one of the most extensively investigated compounds [21,52]. However, to date, such approaches have not been translated into clinical practice and the potential neuroprotective effects of GCase modulators remain unclear, in part because they do not account for *GBA1* mutation type. Therefore, we investigated whether mTOR inhibition affects GCase activity in PBMC-derived macrophages, highlighting a potential therapeutic pathway in GBA1-PD. For the first time, we observed that Torin 1 unexpectedly increased GCase activity in GBA1-PD, regardless of the specific *GBA1* mutation. The precise molecular mechanisms underlying the effects of Torin 1 on GCase activity and lysosome number remain unclear. However, this effect may be mediated through regulation of the transcription factor TFEB, a master regulator of lysosomal biogenesis and autophagy. mTOR kinase functions as an upstream negative modulator of TFEB activity. In turn, TFEB also regulates transcription of the *SCARB2* gene, which encodes LIMP2, the lysosomal transporter responsible for trafficking GCase from the endoplasmic reticulum to lysosomes. Earlier, we demonstrated reduced expression of the *SCARB2* gene in PBMCs from GBA1-PD patients [53], possibly due to altered TFEB activity. Previously, in DA neurons from GBA1-N370S-PD patients, hyperactive mTOR has been shown to cause excessive TFEB phosphorylation and impaired nuclear translocation, whereas pharmacological mTOR inhibition restored TFEB activity, reduced endoplasmic reticulum stress, and decreased alpha-synuclein accumulation [10]. Thus, the observed increase in GCase activity, together with elevated lysosome numbers, may reflect TFEB activation. Taken together, our study demonstrates for the first time that mTOR inhibition enhances GCase activity, highlighting the potential of mTOR inhibitors as a therapeutic strategy for GBA1-PD.

As mentioned above, one of the key functions of mTOR is the regulation of autophagy, which in turn mediates the clearance of aged, misfolded, or overexpressed protein aggregates, including pathological proteins such as alpha-synuclein, as well as damaged organelles. This process begins with the sequestration of a portion of the cytoplasm into a double-membrane vesicle called an autophagosome, which subsequently fuses with a lysosomes to degrade its contents [54]. A crucial step in autophagy is the initiation of autophagosome biogenesis, a process regulated by Beclin-1. We previously demonstrated elevated Beclin-1 protein levels in PBMC-derived macrophages from patients with GBA1-L444P-PD and GBA1-N370S-PD [12], consistent with findings reported in patient-derived cells from GBA1-PD patients carrying different *GBA1* mutations [9,55,56]. Beclin-1 accumulation may result from disrupted ceramide metabolism, endoplasmic reticulum stress, and inflammation, leading to compensatory activation of autophagy [57,58,59,60]. In the present study, Torin 1-mediated mTOR inhibition reduced Beclin-1 levels in PBMC-derived macrophages from both GBA1-N370S-PD and GBA1-L444P-PD patients. The decrease in Beclin-1 upon Torin 1 treatment may reflect modulation of ceramide metabolism, potentially via increased GCase activity. Enhanced GCase function may reduce ceramide levels, while modulation of autophagy may further facilitate their turnover. Recent evidence indicates that mTOR inhibition by Torin 1 induces broad remodeling of lipid metabolism, including sphingolipid pathways and ceramide homeostasis, with reported reductions in ceramide levels [61]. Overall, these findings suggest that changes in Beclin-1 levels are driven not only by autophagy modulation but also by alterations in GCase activity and associated lipid metabolism in GBA1-PD.

Autophagosome formation and turnover are regulated by LC3B and p62. In GBA1-N370S-PD PBMC-derived macrophages, Torin 1 treatment was accompanied by an increased LC3B-II/LC3B-I ratio, enhanced LC3B–lysosome colocalization (which was initially reduced), and decreased p62 levels, suggesting enhanced autophagy. In GBA1-L444P-PD PBMC-derived macrophages, Torin 1 also increased the LC3B-II/LC3B-I ratio and LC3B–lysosome colocalization which was initially reduced, but p62 levels remained unchanged, suggesting incomplete activation of mTOR-dependent autophagy. Similar effects of Torin 1 on autophagic flux have been reported in DA neurons from neuropathic GD patients, with decreased p62, increased LC3B intensity and greater LC3B–LAMP1 colocalization [26], although another study observed no significant changes in p62 or LC3B in GBA1-N370S-PD neurons [10].

Previously, we demonstrated that patients with GBA1-PD exhibit a reduced number of lysosomes in PBMC-derived macrophages [12]. Here, we observed that reduced lysosome number and increased lysosome size are associated with the p.L444P/N mutation in PD. In turn, Torin 1 increased lysosome number in PBMC-derived macrophages from both GBA1-L444P-PD and GBA1-N370S-PD patients, consistent with previous observations in iPSC-derived neurons from GD patients [26,62]. Notably, mTOR inhibition reduced lysosome size in GBA1-L444P-PD PBMC-derived macrophages, which was initially elevated.

This study has several limitations. The sample size of patient-derived macrophages was relatively small, which may limit the generalizability of our findings. α-synuclein levels were not measured, preventing direct assessment of the effects of mTOR inhibition on pathogenic protein accumulation. Autophagy was evaluated without the use of inhibitors such as bafilomycin A1 or chloroquine, or complementary approaches such as flux assays with lysosomal blockade, which would provide a more comprehensive assessment of autophagic activity. In addition, experiments involving the treatment of the neuroblastoma cell line with CBE in combination with the mTOR inhibitor Torin 1, followed by evaluation of mTOR activity, were not performed. Future studies with larger cohorts and additional functional assays are needed to confirm and extend these findings.

## 5. Conclusions

Our study shows that GCase activity increased following mTOR inhibition in PBMC-derived macrophages from GBA1-PD patients regardless of the specific *GBA1* mutation, despite the fact that GBA1-L444P-PD patients exhibit more pronounced GCase deficiency compared to GBA1-N370S-PD. Notably, mTOR inhibition appeared to differentially affect autophagy and lysosome morphology in GBA1-L444P-PD and GBA1-N370S-PD. Torin 1 appeared to more effectively improve autophagy in patients with the «mild» p.N370S mutation. Furthermore, we found that decreased GCase activity and HexSph accumulation are associated with the extent of mTOR kinase activation in vitro. Our findings support a link between GCase activity and mTOR kinase, highlighting mTOR inhibition as a potential therapeutic strategy for GBA1-PD.

## Figures and Tables

**Table 1:** Demographic and clinical characteristics of the compared groups.

Groups	Age at Exam, Mean ± SD, Years	Age at Onset, Mean ± SD, Years	Sex (Female:Male)	Mutations
Full patient cohort				
GBA1-PD, N = 13	58.2 ± 7.7	53.7 ± 7.8	10:3	p.L444P/N, N = 6 p.N370S/N, N = 7
Controls, N = 17	63.5 ± 6.0	-	13:4	-
Patient subgroup included in the mTOR inhibition study				
GBA1-PD, N = 8	60.3 ± 14.3	53.6 ± 12.9	3:5	p.L444P/N, N = 5 p.N370S/N, N = 3

## Data Availability

The original contributions presented in this study are included in the article. Further inquiries can be directed to the corresponding authors.

## Abbreviations

The following abbreviations are used in this manuscript:

PD	Parkinson’s disease
GBA1-PD	PD associated with mutations in the GBA1 gene
GD	Gaucher disease
GlcCer	glucosylceramide
GlcSph	glucosylsphingosine
GBA1-L444P-PD	GBA1-PD carrying the «severe» p.L444P/N mutation
GBA1-N370S-PD	GBA1-PD carrying the «mild» p.N370S/N mutation
PBMCs	peripheral blood mononuclear cells
CBE	conduritol-b-epoxide
GCase	glucocerebrosidase
GALC	galactosylceramidase
GLA	alpha-galactosidase A
acid sphingomyelinase	ASMase
hexosylsphingosine	HexSph
lysosphingomyelin	LysoSM
lysoglobotriaosylsphingosine	LysoGb3
iPSC	induced pluripotent stem cells
DA	dopaminergic
